# Synthesis and Characterization of Nitric Oxide-Releasing Ampicillin as a Potential Strategy for Combatting Bacterial Biofilm Formation

**DOI:** 10.1021/acsami.3c00140

**Published:** 2023-03-16

**Authors:** Lori M. Estes Bright, Mark Richard Stephen Garren, Megan Douglass, Hitesh Handa

**Affiliations:** † School of Chemical, Materials, and Biomedical Engineering, University of Georgia, Athens, Georgia 30602, United States; ‡ Pharmaceutical and Biomedical Sciences Department, College of Pharmacy, University of Georgia, Athens, Georgia 30602, United States

**Keywords:** nitric oxide, antibiotic, antimicrobial, *S*-nitroso-*N*-acetylpenicillamine, ampicillin

## Abstract

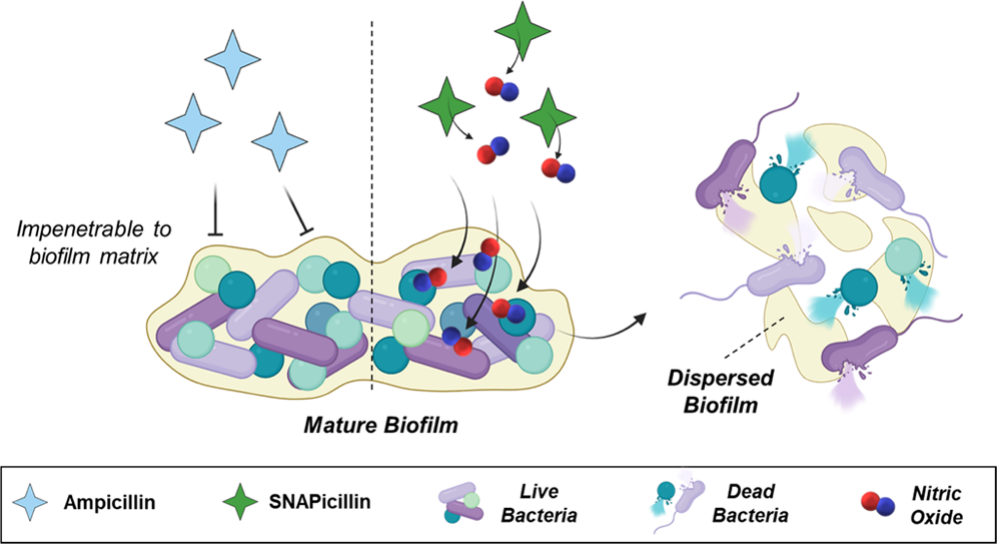

Biofilm formation on biomaterial interfaces and the development of antibiotic-resistant bacteria have decreased the effectiveness of traditional antibiotic treatment of infections. In this project, ampicillin, a commonly used antibiotic, was conjugated with *S*-nitroso-*N*-acetylpenicillamine (SNAP), an *S*-nitrosothiol compound (RSNO) used for controlled nitric oxide (NO) release. This novel multifunctional molecule is the first of its kind to provide combined antibiotic and NO treatment of infectious pathogens. Characterization of the molecule included NMR, FTIR, and mass spectrometry. NO release behavior was also measured and compared to pure, unmodified SNAP. When evaluating the antimicrobial efficacy, the synthesized SNAPicillin molecule showed the lowest MIC value against Gram-negative *Pseudomonas aeruginosa* and Gram-positive methicillin-resistant *Staphylococcus aureus* compared to ampicillin and SNAP alone. SNAPicillin also displayed enhanced biofilm dispersal and killing of both bacterial strains when treating a 48 h biofilm preformed on a polymer surface. The antibacterial results combined with the biocompatibility of the molecule show great promise for infection prevention and treatment of polymeric interfaces to reduce medical device-related infections.

## 1 Introduction

Dangerous infections on medical device surfaces and implants begin with the attachment of a single bacterium. If left unchecked, more bacteria locally adhere and begin the process of biofilm formation, characterized by the production of an extra polymeric substance composed of polysaccharides, extracellular DNA, and secreted proteins. The EPS acts as a protective barrier against the environment and allows bacteria to remain in a semidormant state, less vulnerable to metabolic attack.^1,2^ Unfortunately, traditional antibiotic treatments are ineffective against biofilms, as the bulky molecules are unable to penetrate the EPS matrix, leaving bacteria to further multiply and biofilms to mature.^2^ In fact, studies have shown that it can take antibiotic concentrations up to 1000× the standard dose to even have a minute effect on biofilm viability.^3,4^ However, these high concentrations of antibiotics contribute to the development of antibiotic-resistant bacteria that are then more difficult to treat if they are not fully eradicated and can be fatal. For instance, in the US and Europe, it has been found that methicillin-resistant *Staphylococcus aureus* (MRSA) is responsible for the deaths of over 50,000 people per year.^5^ Aside from the rise in mortality rates, antibiotic resistance can also have severe financial impacts. By the year 2050, antimicrobial-resistant infections will not only cause 10 million deaths per year but will also lead to a total GDP loss of over $100 trillion if solutions are not found.^5^ The issues associated with difficulties in biofilm treatment have led the biomedical community to seek alternative and combination therapies that can provide some relief from the onslaught.

Nitric oxide (NO) gas has recently emerged as an effective antimicrobial treatment in research. Endogenously, NO is utilized in many physiological roles including as a neurotransmitter and vasodilation agent when it is released by endothelial cells in blood vessels.^6^ Most importantly, it is released by macrophages as a part of the immune system to fight off foreign pathogens.^7^ NO is a free radical gas, allowing for the creation of potent reactive oxygen species (ROS) and reactive nitrogen species (RNS) through side reactions with environmental molecules. NO, along with these ROS and RNS induce oxidative and nitrosative stress on bacterial cells, killing microbes with no chance for resistance development.^8−10^ In fact, a prior study showed that, even after systematic and repeated exposure to NO-releasing particles, *Staphylococcus aureus*, MRSA, *Staphylococcus epidermidis*, *Escherichia coli*, and *Pseudomonas aeruginosa* (*P. aeruginosa*) showed no increase in MIC values, indicating a lack of ability of these bacteria to develop NO resistance by spontaneous mutagenesis.^11^ NO has also shown antimicrobial activity against viruses, parasites, and fungi in previous studies.^8,10,12^ However, due to the instability of the gas molecule alone, synthetic NO donor molecules have been developed for integration into biomedical systems that release NO under specific conditions.^13^*S*-nitrosothiols, or RSNOs, are one of the most popular NO donors used in biomedical applications, as they release NO in response to light, enzymes, metal ions, and heat, including levels characteristic of physiological conditions (37 °C).^14^

Many studies have shown the antimicrobial potential of RSNO incorporation into medical devices and polymer coatings. Vascular^15,16^ and urinary catheters^17,18^ have been modified, as well as PVC endotracheal tubes^19^ and sol–gel coatings for medical grade materials,^20,21^ all showing greater antibacterial capacity compared to control surfaces. Antibiotic delivery from a polymer system has also shown antibacterial success,^22^ but the superior combination treatment of NO donor molecules and antibiotics has led to biofilm disruption and bacterial killing, as bacteria are more vulnerable to treatment with traditional antibiotics following interaction with NO.^23−25^

The success of NO in biofilm dispersal has recently been investigated in *P. aeruginosa* biofilms. Results from the study showed that NO-mediated biofilm dispersal draws back to the activity of the intracellular second messenger cyclic di-GMP (c-di-GMP). Low levels of exogenous NO release lead to upregulation of a specific phosphodiesterase (PDE) that degrades c-di-GMP, triggering more motility of bacteria and cell dispersal.^26^ This regulation leads to a more planktonic state of bacteria from biofilms, which has been demonstrated in multiple studies following NO treatment.^27−30^ One study even showed that NO release levels as low as 450 pM can lead to biofilm dispersal of *P. aeruginosa*, inducing higher susceptibility to successive antibiotic treatment.^31^ However, NO release at that low level is unable to completely eradicate bacterial colonization on infected surfaces, and a combination therapy must be employed. Fortunately, previous studies have shown that sequential treatment of bacteria with NO, followed by antibiotics increased the susceptibility of the bacteria to antibiotic therapy and decelerated the development of antibiotic resistance.^32^ The success of NO in biofilm penetration combined with a known potent antibiotic may ensure complete obliteration of an infection. These results are promising but still require multiple antimicrobial treatment stages. Moreover, a multifunctional antimicrobial agent that can be easily incorporated into different polymeric substrates can help prevent biofilm formation on medical device surfaces in clinical settings.

Herein, we covalently attach a synthetic RSNO, *S*-nitroso-*N*-acetylpenicillamine (SNAP), to ampicillin to create a novel dual-functional antimicrobial agent (SNAPicillin). This is the first study employing RSNO chemistry to combine NO and antibiotic therapies into a single molecule. By combining NO and ampicillin into one compound, biofilm dispersal by NO can then lead to higher susceptibility of bacteria to antibiotic treatment. The synthesized molecule was verified and characterized via ^1^H and ^13^C nuclear magnetic resonance (NMR), Fourier transform infrared (FTIR) spectroscopy, electrospray ionization mass spectroscopy (ESI-MS), functional handle quantification, and NO release studies via chemiluminescence-based detection of NO via a Zysense nitric oxide analyzer (NOA 280i). The antimicrobial efficacy of SNAPicillin was then evaluated both in the solution phase and on a biofilm. First, minimum inhibitory concentrations (MICs) were determined against Gram-negative *Pseudomonas aeruginosa* (*P. aeruginosa*) and Gram-positive methicillin-resistant *Staphylococcus aureus* (MRSA). Then, biofilm dispersal and bacterial elimination were evaluated against 48 h mature biofilms grown onto polymeric films of CarboSil, a biocompatible copolymer composed of polycarbonate-urethane with silicone. Lastly, the biocompatibility of the molecule was evaluated against 3T3 mouse fibroblast cells at the antimicrobial-relevant concentrations.

## 2 Materials and Methodology

### 2.1 Materials

Acetic anhydride, anhydrous magnesium sulfate, chloroform, ethanol, ethylenediaminetetraacetic acid (EDTA), glutaraldehyde, hexamethyldisilazane (HMDS), hexanes, hydrochloric acid, methanol, Mueller–Hinton broth and agar (MHB and MHA), *N*-acetylpenicillamine, *N,N*-dimethylformamide-d7, phosphate-buffered saline (PBS), pyridine, sulfuric acid, *t*-butyl nitrite, tetrahydrofuran (THF), and 3-[4,5-dimethylthiazol-2yl]-2,5-diphenyl-tetrazolium bromide (MTT) were purchased from Sigma-Aldrich (St. Louis, MO). Sodium ampicillin was purchased from Gold Biotechnology (St. Louis, MO). Gram-positive methicillin-resistant *Staphylococcus aureus* (MRSA, ATCC BAA 41) and Gram-negative *Pseudomonas aeruginosa* (*P. aeruginosa* ATCC 9027) were obtained from American Type Culture Collection (ATCC, Manassas, VA).

### 2.2 SNAPicillin Synthesis

**Figure 1:**
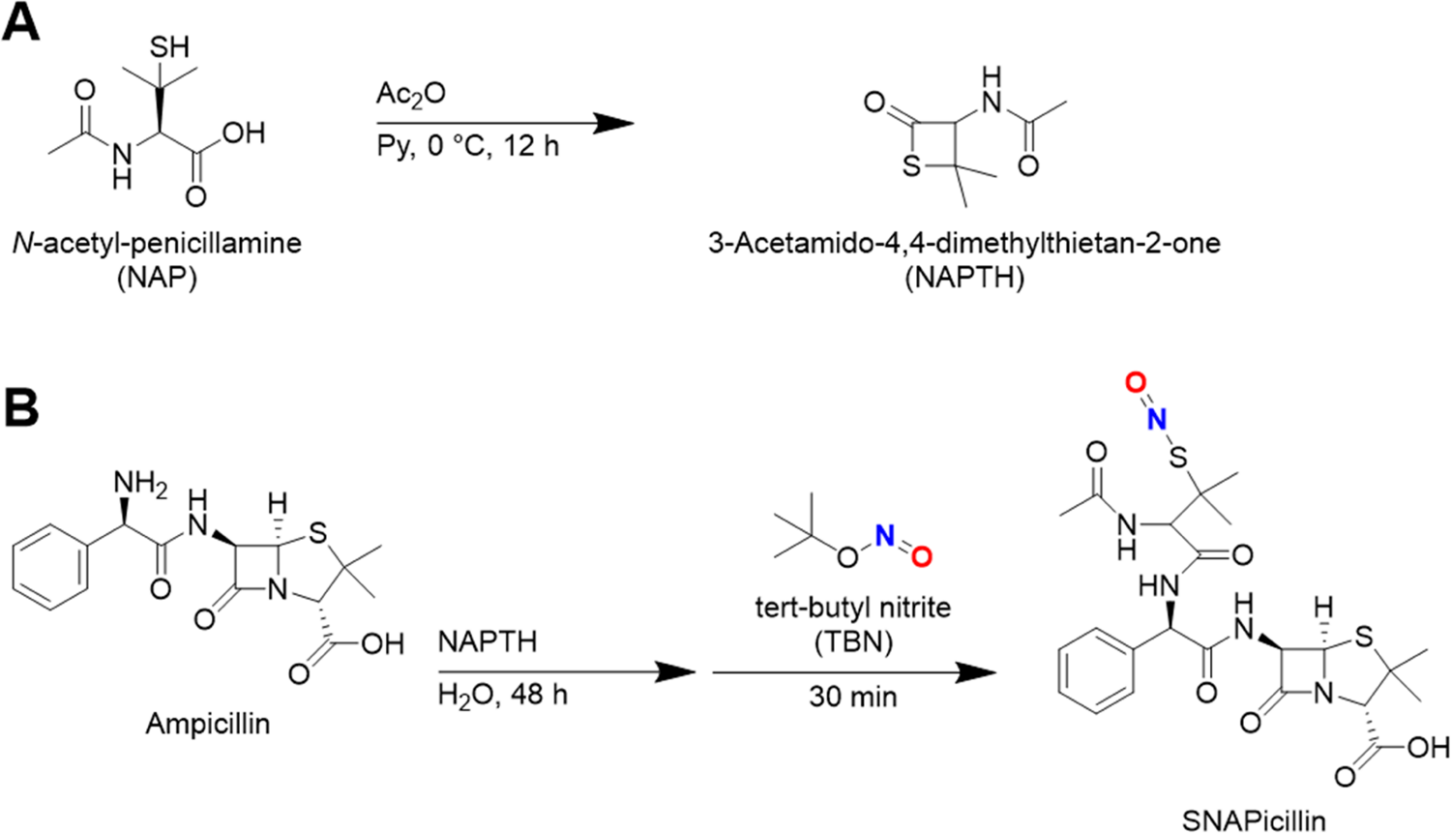
SNAPicillin preparation via the sequential reaction of ampicillin with a thiolactone derivative of *N*-acetyl-penicillamine and nitrosation. (A) Synthesis of *N*-acetyl-penicillamine thiolactone (NAPTH). (B) Covalent immobilization of the NO donor group via thiolactone conjugation of ampicillin, followed by organic nitrosation.

Covalent immobilization of a tertiary RSNO pendant group to ampicillin was achieved via indirect conjugation of *N*-acetyl-d-penicillamine (NAP) via its thiolactone derivative (Figure 1A). The self-protected thiolactone of NAP, hereafter referred to as 3-acetamido-4,4-dimethylthietan-2-one (NAPTH), was prepared following previously reported methods with minor deviation.^33,34^ NAP (5 g, ∼26 mmol) was dissolved in ice-cooled, anhydrous pyridine (Py, 20 mL). Ice-cooled acetic anhydride (Ac_2_O, 9.26 g, ∼90 mmol) was then added to the mixture. The mixture was reacted for 12 h; afterward, the solvent was removed by rotary evaporation, and the crude was resuspended in chloroform (30 mL). The organic phase was washed three times with an equal volume of HCl (1 M) and dried over magnesium sulfate. The organic phase was reduced and triturated in hexanes overnight. The resulting crystalline white powder was collected via vacuum filtration and dried under vacuum overnight.

Attachment of the NAP pendant group to ampicillin was achieved via aminolysis of the self-protected NAPTH (Figure 1B). Ampicillin sodium salt (1 g, ∼2.7 mmol) was combined with an equimolar amount of NAPTH (466 mg) in deionized water (32 mL). The pot was stirred for 48 h at room temperature. Afterward, the pot was chilled and *t*-butyl nitrite in molar excess (1.7 g, ∼16.8 mmol) was added dropwise. The crude SNAPicillin product formed with significant frothing. Additional chilled deionized water (10 mL) was added slowly to aid precipitation. CAUTION:*Addition of deionized water to the froth can facilitate the release of nitrogen oxide (NO_x_) gases from the reaction pot. Only perform this reaction in a fully functioning fume hood running at optimal capacity.*

Finally, the SNAPicillin product was vacuum-filtered and washed three times with chilled deionized water (10 mL) and once with chilled ethanol (10 mL) before being dried under vacuum overnight. The SNAPicillin product was stored at −20 °C with protection from light.

### 2.3 SNAPicillin Characterization

#### 2.3.1 NMR/FTIR/MS Characterizations

Compounds were assessed via Fourier transform infrared spectroscopy (FTIR) using a Spectrum 3 spectrometer with a KBr pellet loading method (PerkinElmer, Greenville, SC). Pellets were assessed for absorbance in the region of 4000–650 cm^–1^ over a total of 128 scans with a resolution of 4 cm^–1^. Compounds were further characterized via nuclear magnetic resonance (NMR) spectroscopy using a spectrometer equipped with an Ascend 400 MHz magnet and Advance III HD Nanobay console (Bruker, Billerica, MA). Compounds were scanned in methanol-d_4_ for a total of 64 scans for ^1^H experiments and 1024 scans for ^13^C experiments. Finally, routine mass spectra (MS) were obtained on a Bruker Esquire 3000 Plus Ion spectrometer in positive ion mode using direct sample injection in methanol.

#### 2.3.2 Degree of Conjugation

Ellman’s test for thiols was used to determine the degree of conjugation of NAP-thiolactone to the primary amine present in ampicillin.^35^ In short, stock solutions of 5,5′-dithio-bis(2-nitrobenzoic acid) (DTNB) and sodium acetate were prepared in DI water at concentrations of 2 and 50 mM, respectively. The combination of 50 μL of the DTNB solution, 100 μL of Tris buffer (1 M, pH = 8.0), 840 μL of deionized water, and 10 μL of a NAP-ampicillin solution of a known concentration formed the working reagent. Following incubation of the solution for 15 min at room temperature, the absorbance was measured at 412 nm using a Genesis 10S UV–vis spectrophotometer. The absorbance was divided by the extinction coefficient of the reagent (13,600 M^–1^ cm^–1^)^36^ to calculate the molarity of thiol functionality. The assay was calibrated using cysteine to form a standard curve of thiols.

Once conjugation efficiency was quantified, the nitrosation efficiency of the exposed thiol was determined by inducing catalytic decomposition of the RSNO group and NO quantification using the NOA. A catalytic solution of 30 μL of 50 mM CuCl_2_, 1.5 μL of 10 mM cysteine, and 2945 μL of PBS was added to the amber reaction chamber. A SNAPicillin solution of known concentration was injected into the chamber, and NO release was measured to exhaustion. The actual amount of NO released was then compared to the theoretical amount of NO loaded if all present thiols were nitrosated and efficiency (%) was calculated.

#### 2.3.3 Nitric Oxide Release Kinetics

NO release was measured using chemiluminescent detection methods using a Sievers 290i nitric oxide analyzer (NOA), the gold standard in NO quantification. SNAP and SNAPicillin samples were prepared by dissolving the molecules in 10 mM PBS with 100 μM EDTA. EDTA is used to chelate any metal ion impurities present in the DI water to prevent the catalytic decomposition of the RSNO bonds. Samples were measured in an amber reaction vessel to prevent light activation and subsequent NO release from the molecules and maintained at 37 °C using a water bath to represent physiological conditions. A continuous stream of nitrogen gas at 200 mL min^–1^ was used to purge NO from the reaction chamber and into the NOA where the following reaction took place.
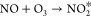

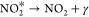
NO reacted with ozone to produce NO_2_*, which releases a photon upon dropping back down to the ground state. This photon is measured by the instrument, converted to a voltage signal, and then converted to a PPB or PPM reading. A calibration constant specific to the NOA is then used to convert PPB readings into a mol min^–1^ mL^–1^ NO release value. The NO release from the samples was measured at 0 h and 24 h, and samples were stored in a 37 °C incubator between readings.

### 2.4 Antibacterial Efficacy of SNAPicillin

#### 2.4.1 Minimal Inhibitory Concentration (MIC) Determination

The minimum inhibitory concentrations (MICs) for Ampicillin, SNAP, and SNAPicillin were determined in a 24 h bacterial growth study against *P. aeruginosa* and MRSA. Concentrations of all three treatments ranged from 1.91 nM to 8 mM. An initial stock solution of 16 mM was made, and treatment concentrations were diluted by half down to the final treatment. The bacterial solution was prepared by first inoculating a single colony into MHB media and incubating at 37 °C and 150 rpm until the log phase of growth was reached. At that point, the bacterial suspension was collected, rinsed with PBS, and resuspended in MHB. The absorbance of the solution was read at 600 nm, and the bacterial counts were adjusted to ∼10^7^ CFU mL^–1^. Once both the treatment and bacterial solutions were prepared, 75 μL of treatment and 75 μL of bacteria were added to the appropriate wells of a 96-well plate. Initial absorbances were taken to maintain a proper experimental setup; then, the plates were sealed with parafilm, covered with aluminum foil, and placed in a shaker incubator at 37 °C and 150 rpm for 24 h. Following 24 h of bacterial growth, the absorbance of the well plate was read at 600 nm. The final absorbances of the growth curves were plotted against treatment concentration. Blanks for media and each treatment were subtracted for analysis, and *n* = 4 wells were analyzed per treatment. Experiments were carried out in biological triplicates to confirm results.

#### 2.4.2 Biofilm Reduction Assay

Biofilms were grown for 48 h on 8 mm diameter CarboSil films and then treated with ampicillin, SNAP, and SNAPicillin solutions. Solutions of *P. aeruginosa* and MRSA were prepared by placing one colony of bacteria in MHB and incubating at 37 °C and 150 rpm until the solution reached approximately 10^6^ to 10^8^ CFU mL^–1^. The bacteria pellet was collected, rinsed with PBS, resuspended in MHB media, and further diluted 1:100 in MHB media. Following dilution, 1 mL of bacterial solution was added to each well of a 48-well plate containing a sterilized 8 mm diameter CarboSil film. The plate was sealed with parafilm, covered with aluminum foil, and placed in an incubator at 37 °C for 48 h. At 24 h, the medium was removed and replaced with fresh MHB media to replenish nutrients without disturbing biofilm growth. After 48 h of growth, films were removed from the wells, rinsed lightly with PBS, and placed in a new 48-well plate. Solutions of ampicillin, SNAP, and SNAPicillin were then added to the wells at a volume of 1 mL. Control films were treated with PBS. *P. aeruginosa* biofilms were treated with 250 μM, and MRSA biofilms were treated with 2 mM of each treatment, per results from the MIC studies. The plate was then placed in a shaking incubator for 24 h at 150 rpm and 37 °C. Following treatment, films were rinsed lightly in PBS, then homogenized and vortexed for 1 min each to remove any adhered bacteria and biofilm. Bacterial solutions were then diluted and plated on LB agar plates and placed in an incubator for ∼18 to 24 h, and CFUs were counted. The bacterial killing was normalized for the surface area of the CarboSil films, and *n* = 4 films were used per treatment.

To visualize biofilm dispersal, the experiment was repeated, but after 24 h of treatment, the CarboSil films were removed from solution, lightly rinsed in PBS, and placed in 3% glutaraldehyde to fix the attached biological mass. Samples were then dehydrated using increasing ethanol concentrations, followed by final drying by HMDS. Films were affixed to scanning electron microscopy (SEM) stubs and sputter-coated with 10 μm gold-palladium using a Leica sputter coater (Leica Microsystems). Images were acquired using an SEM (FEI Teneo, FEI Co.) setup at an accelerating voltage of 5.00 kV.

### 2.5 Biocompatibility Evaluation of SNAPicillin

The biocompatibility of SNAPicillin as well as its ampicillin and SNAP analogues was assessed using an *in vitro* cytotoxicity assay with human fibroblast cells (BJ CRL-2522) in accordance with ISO-10993–5 standards.^37^ Fibroblast cells were revived from cryopreserved stocks and cultured in DMEM media supplemented with 10% FBS and 1% S/P at 37 °C in a 5% CO_2_-humidified incubator. Cells were cultured for up to 10 passages, with cells split once >70% subconfluency was reached. For cytocompatibility experiments, cells were detached with 0.05% trypsin supplemented with 5 mM EDTA, collected via centrifugation, counted, plated in 96-well tissue culture-treated plates (5000 cells/well), and grown for 24 h to reach ∼70% monolayer confluency.

Biocompatibility was screened through direct contact testing of compounds against fibroblast cells. Following cell seeding and 24 h incubation, media in wells was replaced with clean media supplemented with various concentrations of ampicillin, SNAP, and SNAPicillin. Cells were grown for an additional 24 h, after which the medium was again decanted and cells were washed with PBS (1×). Cells were then incubated in PBS supplemented with 0.5 mg mL^–1^ MTT reagent. Cells were grown for an additional 3 h and read for absorbance at 570 nm with a reference reading at 690 nm. Relative cell viability was then calculated from the change in absorbance reading, Δ_ABS 570–690_, with respect to untreated cells as follows
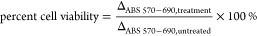
Final data are reported as mean percent cell viability ± standard deviation (*n* = 3 independent passages).

### 2.6 Statistical Analysis

Data obtained for each performed characterization study are expressed as mean ± standard deviation (SD). Statistical analysis was performed on NOA studies using one-way ANOVA at each time point. Antimicrobial and biocompatibility studies were analyzed for statistical significance using one-way ANOVA with Tukey post hoc correction and two-way ANOVA, respectively. *P*-values < 0.05 were considered statistically significant for all experimental results.

## 3 Results and Discussion

### 3.1 Synthesis and Characterization of Covalently Attached SNAP to Ampicillin (SNAPicillin)

Pairing NO donors and related compounds (e.g., *N*-diazeniumdiolates, nitrite, and nitroxides) with μg/mL and sub μg/mL concentrations of conventional antibiotics (e.g., chloramphenicol, ciprofloxacin, gentamicin, tetracycline, tobramycin, etc.) better potentiates antibiotic effects by decreasing tolerance and slowing antibiotic resistance development.^23,24,32^ Despite the demonstrated therapeutic efficacy of combining NO with conventional antibiotics, codelivery can be inherently challenging in a clinical setting and presents further logistical challenges in scheduling and standardized dosing times. By conjugating a NO-donating SNAP moiety to conventional antibiotics, both the therapeutic benefits of NO and the conventional antibiotic may be delivered concomitantly for localized effect against both planktonic bacteria and biofilms. Furthermore, incorporation of an RSNO group in lieu of *N*-diazeniumdiolates or other NO donors offers the potential for improved controlled release of NO, as RSNOs have shown improved stability over *N*-diazeniumdiolates and many other NO donors under physiological conditions.^38,39^ Contemporary application of RSNOs such as *S*-nitrosoglutathione (GSNO), SNAP, and *S*-nitroso-*N*-acetyl-*L*-cysteine ethyl ester (SNACET) in lock solutions and other therapies has shown their utility for developing prolonged, stable release of NO under physiological conditions, unlike *N*-diazeniumdiolate counterparts, which exhibit cytotoxic levels of NO burst release with additional cytotoxicity concerns from polyamine byproduct formation.^40−42^

**Figure 2:**
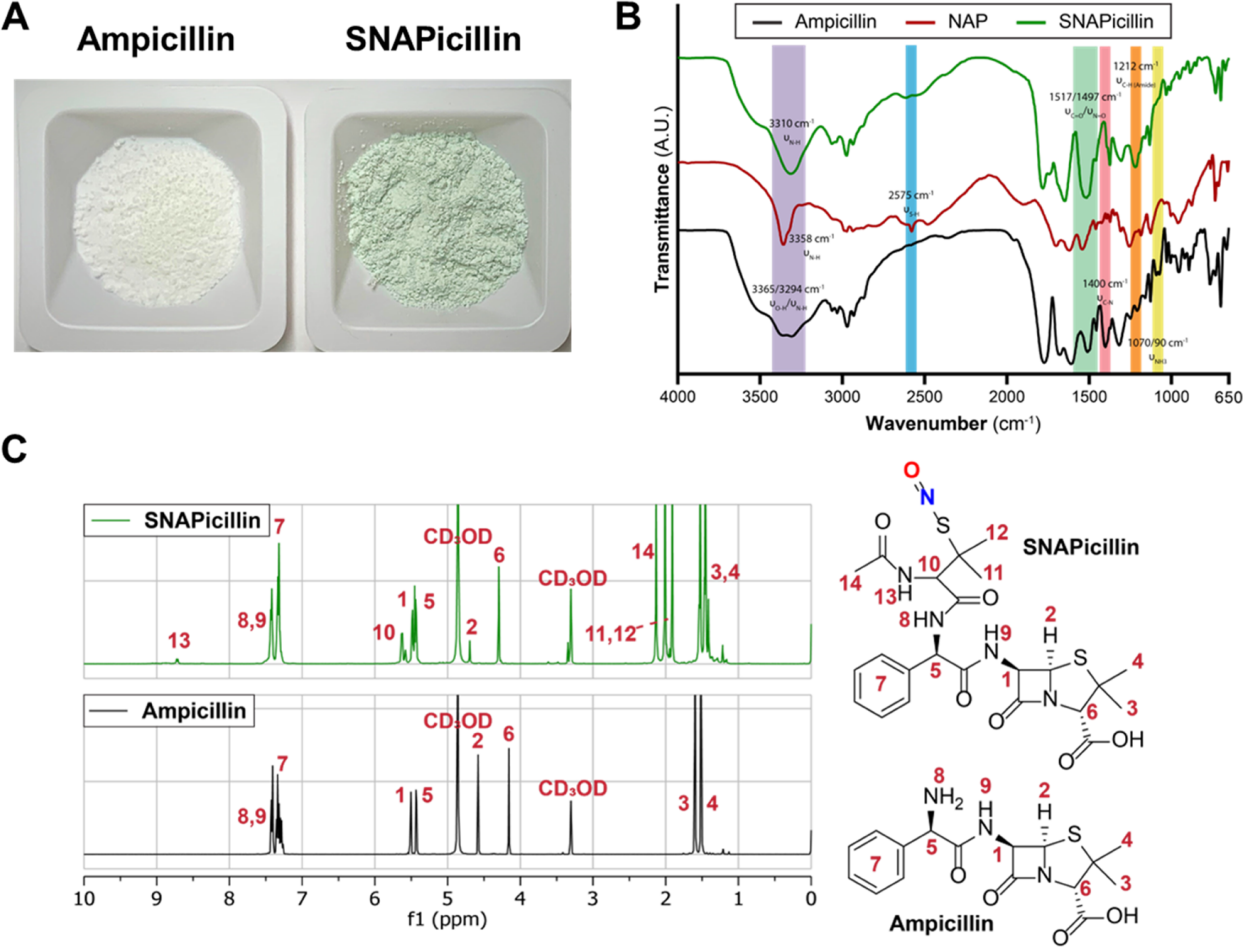
SNAPicillin was readily precipitated as (A) a green powder. (B) FTIR characterization of SNAPicillin powder supported the presence of the RSNO group through characteristic bond vibration (1497 cm^–1^) as well as other characteristic peaks consistent with the NO donor precursor NAP. (C) ^1^H NMR spectroscopy (400 MHz, CD3OD) of SNAPicillin further demonstrated bond formation and conjugation of NAP to ampicillin.

Herein, covalent conjugation of the tertiary RSNO NO donor SNAP to the clinically ubiquitous, broad-spectrum antibiotic ampicillin is performed to form a novel therapy with SNAPicillin. The SNAPicillin product readily precipitated as a green substrate (Figure 2A). Key functional groups on SNAPicillin were verified via FTIR (Figure 2B) and ^1^H and ^13^C NMR spectroscopy (Figures 2C and S1). FTIR analyses of the isolated SNAPicillin product showed the emergence of several bands characteristic of the NO donor precursor, NAP, as well as N=O stretching vibrations characteristic of RSNOs (1497 cm^–1^). Further ^1^H NMR studies of the isolated SNAPicillin supported aminolysis of NAPTH with ampicillin’s amine via evidence of ring-opening and the presence of several distinct hydrogen environments associated with NAP’s amide group (labels 13 and 14) and its chiral center (label 10, see Figure 2C). ^13^C NMR studies further supported these findings, showing distinct carbon environment formation in the final isolated product (Figure S1). Finally, further mass spectrometry studies demonstrated two peaks at ∼555.0 and 573.8 *m*/*z* ratios, corresponding to decomposed SNAPicillin with released NO as well as pristine SNAPicillin (551.63 g/mol). The solubility of SNAPicillin decreased when compared to its ampicillin precursor in PBS. While ampicillin shows solubility up to ∼50 mg mL^–1^, SNAPicillin displayed solubility up to ∼5 mg mL^–1^ utilizing vigorous stirring. However, this is improved upon the SNAP alternative, at ∼2 mg mL^–1^ in PBS. These enhanced solubility limits for SNAPicillin afford it greater opportunity in aqueous settings than previously possible with SNAP.

The synthetic route to SNAPicillin was further monitored for reaction efficacy via sulfhydryl quantification with Ellman’s assay. The initial thiolactone aminolysis step facilitating ring-opening and binding to the terminal amine group of ampicillin was quantified by the total amount of free sulfhydryl formed. Using this assay, the primary conversion of primary amine sites to NAP functional groups was found to be 86.9 ± 0.6%. Prior studies of β-thiolactones have shown comparable aminolysis conversion efficacies, with steric hindrance leading to reduced pseudo-first-order reaction rates.^43^ By extending the aminolysis reaction over 48 h in stoichiometric excess of NAPTH, we found our optimal conversion at 86.9% amine conversion of ampicillin to its thiol conjugate. The amine group on ampicillin is neighbored by both an electrophilic carbonyl group and a nucleophilic aromatic center (Figure 1), leading to a center suitable for nucleophilic ring-opening and subsequent thiol functionalization. Following nitrosation of the crude mixture, precipitation, and workup, the total conversion of thiols to RSNO groups was found to be in excess of 98.7 ± 1.5% via catalytic NOA analysis with copper(II) chloride and l-cysteine, demonstrating a high degree of nitrosation efficiency and purity corroborated by NMR and MS studies. The ∼85.77% yield of SNAPicillin from ampicillin through this modification pathway demonstrates the facile preparation of the SNAPicillin conjugate and supports further study of its NO-releasing kinetics.

### 3.2 Nitric Oxide Release Kinetics

**Figure 3:**
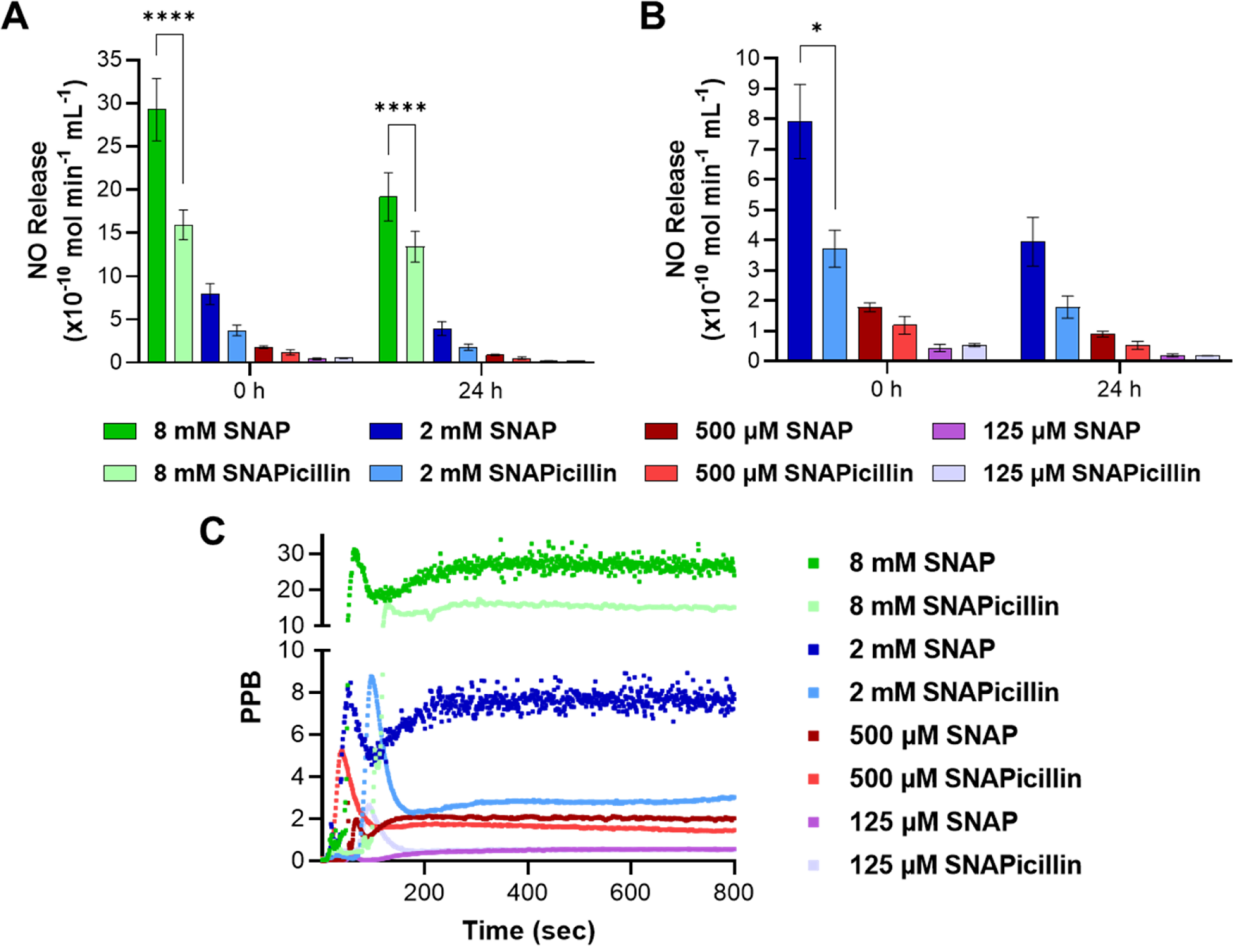
(A, B) Nitric oxide release from SNAPicillin was compared to SNAP over a 24 h period. (C) Instantaneous NO measurements showed stabilized release after ∼150 to 200 s. Statistical significance is denoted as * (*p* < 0.05) and **** (*p* < 0.0001).

The NO released from SNAPicillin was measured using chemiluminescent detection methods and compared to SNAP alone. Solutions of SNAP and SNAPicillin were made by dissolving equimolar amounts of the two compounds in PBS (pH = 7.4) with EDTA as a chelating agent. Physiological conditions of the reaction chamber were maintained at 37 °C using a water bath, and NO release by thermal degradation was measured in real-time. The solutions of SNAP showed a slightly higher average NO release compared to SNAPicillin solutions over a 24 h period (Figure 3A,B and Table S1). However, both SNAP and SNAPicillin showed a stable real-time NO release profile (Figure 3C). The NO release from SNAPicillin followed the same behavior as SNAP, previously found to be pseudo-first-order, where the decomposition is proportional to the available concentration.^44^ Although both compounds display NO release only lasting a few days, stability can be improved through impregnation into a polymer matrix, delaying hydrolysis and prolonging the NO lifetime. Further, although equimolar amounts of each component were used, the RSNO component on the SNAPicillin appears to be less reactive when compared to pure crystalline SNAP under physiological conditions. This follows a general trend in RSNO reactivity where larger molecules have increased intramolecular interactions that enhance the overall stability of the compound.^45^ This demonstrates that SNAPicillin has the potential to prolong the NO release duration as an administered solution and potentially when incorporated into polymer matrices.

### 3.3 Antibacterial Efficacy of SNAPicillin

#### 3.3.1 Minimal Inhibitory Concentration (MIC) Determination

**Figure 4:**
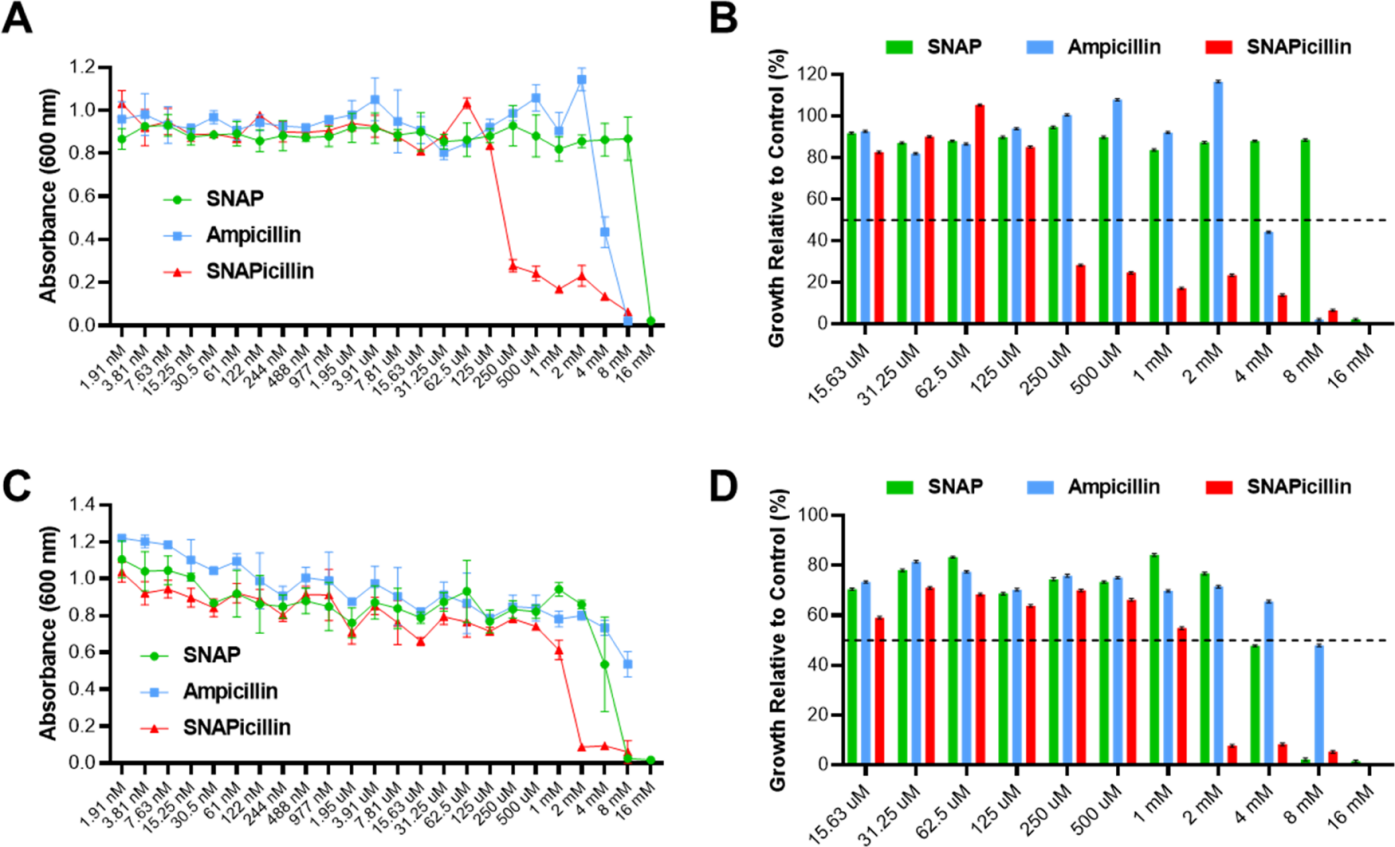
(A) *P. aeruginosa* MIC assay revealed that SNAPicillin showed the greatest antimicrobial activity after 24 h treatment, (B) with an MIC_50_ value of 250 μM. (C) MRSA MIC assay displayed comparable efficacy, with SNAPicillin showing the highest antimicrobial abilities, (D) but with a higher SNAPicillin concentration of 2 mM showing greater than 50% bacterial killing compared to controls, where ampicillin and SNAP were both ineffective.

The MIC of the new SNAPicillin molecule was evaluated to determine the antibacterial efficacy of the compound compared to ampicillin, a commonly used antibiotic that we chose to modify, and SNAP, the NO donor utilized for modification. Bacterial growth of Gram-negative *P. aeruginosa* and Gram-positive MRSA was measured through the absorbance values of bacterial solutions in a 96-well plate over 24 h. Following 24 h of treatment, absorbances were plotted versus treatment concentration. MIC_50_ values were calculated based on the concentration needed to reduce growth relative to controls by 50%. Treatment of *P. aeruginosa* revealed that SNAPicillin showed growth reduction at lower treatment concentrations than both ampicillin and SNAP (Figure 4A). When the bacterial growth was compared to the control wells, it was shown that the MIC_50_ value for SNAPicillin is 250 μM, whereas that for ampicillin is 4 mM and SNAP is 16 mM (Figure 4B). These values were confirmed with two additional biological replicates (Figure S2). SNAPicillin showed an MIC_50_ value of 125 μM in the two replicate studies, but 250 μM was chosen as the MIC_50_ concentration since it was the lowest concentration to show >50% killing in all three studies. For the treatment of MRSA, all three compounds showed a similar trend, with SNAPicillin displaying the highest antimicrobial efficacy at the lowest concentration (Figure 4C). SNAPicillin had an MIC_50_ value of 2 mM, while SNAP was 4 mM and ampicillin did not show >50% killing until 8 mM treatment (Figure 4D). These results are not surprising as the *S. aureus* bacterial strain we tested is methicillin-resistant. Both methicillin and ampicillin are β-lactam antibiotics that bind to penicillin-binding proteins involved in bacterial cell wall synthesis.^46^ Therefore, it is reasonable to assume that the MRSA used in our study has potentially developed β-lactamase enzymes that deactivate the antimicrobial activity of ampicillin. The MIC_50_ results for MRSA were also confirmed with two additional biological replicates (Figure S3). Additionally, SNAP alone was not effective against either *P. aeruginosa* or MRSA over 24 h until concentrations of 4–16 mM were reached, a finding that is consistent with previous antimicrobial studies of SNAP in solution.^47^ Only the combination of SNAP and ampicillin in our newly synthesized molecule, SNAPicillin, was effective as an antimicrobial agent in solution. Again, these combination-treatment results are consistent with the previous literature that has shown that combining NO-releasing moieties with traditional antibiotics decreases bacterial tolerance to antimicrobial therapies and also slows the development of antibiotic resistance if it were to occur.^23,24,32^

#### 3.3.2 Biofilm Reduction Assay

The antibacterial success of SNAPicillin against bacteria in solution is promising for the biomedical world, though there is a more pressing issue facing the world of infection. Biofilms are composed of bacterial colonies surrounded by a protective layer of extra polymeric substances (EPSs) composed of polysaccharides, extracellular DNA, and secreted proteins.^1,2^ This EPS acts as a protective barrier against antibiotics, as they are unable to penetrate the polymeric matrix. Further, bacteria in biofilms are often in a metabolically dormant state, necessitating fewer nutrients for survival and much higher concentrations of antibiotics, often 1000× higher, to initiate bacterial killing.^3^ This limitation of antibiotics can be combatted by a dual-antimicrobial activity compound such as SNAPicillin. The hypothesized effectiveness of SNAPicillin against biofilms lies in the gaseous nature of NO, shown in previous studies to disperse the biofilm matrix, causing internalized bacteria to be more susceptible to antimicrobial treatments.^27,48^ Thus, the proposed anti-biofilm mechanism of SNAPicillin is twofold: (1) initial NO gas released from the bound-RSNO group disperses the biofilm matrix, and then (2) NO and ampicillin work in conjunction to kill bacteria that are no longer protected by the biofilm EPS, leading to enhanced killing compared to ampicillin alone.

**Figure 5:**
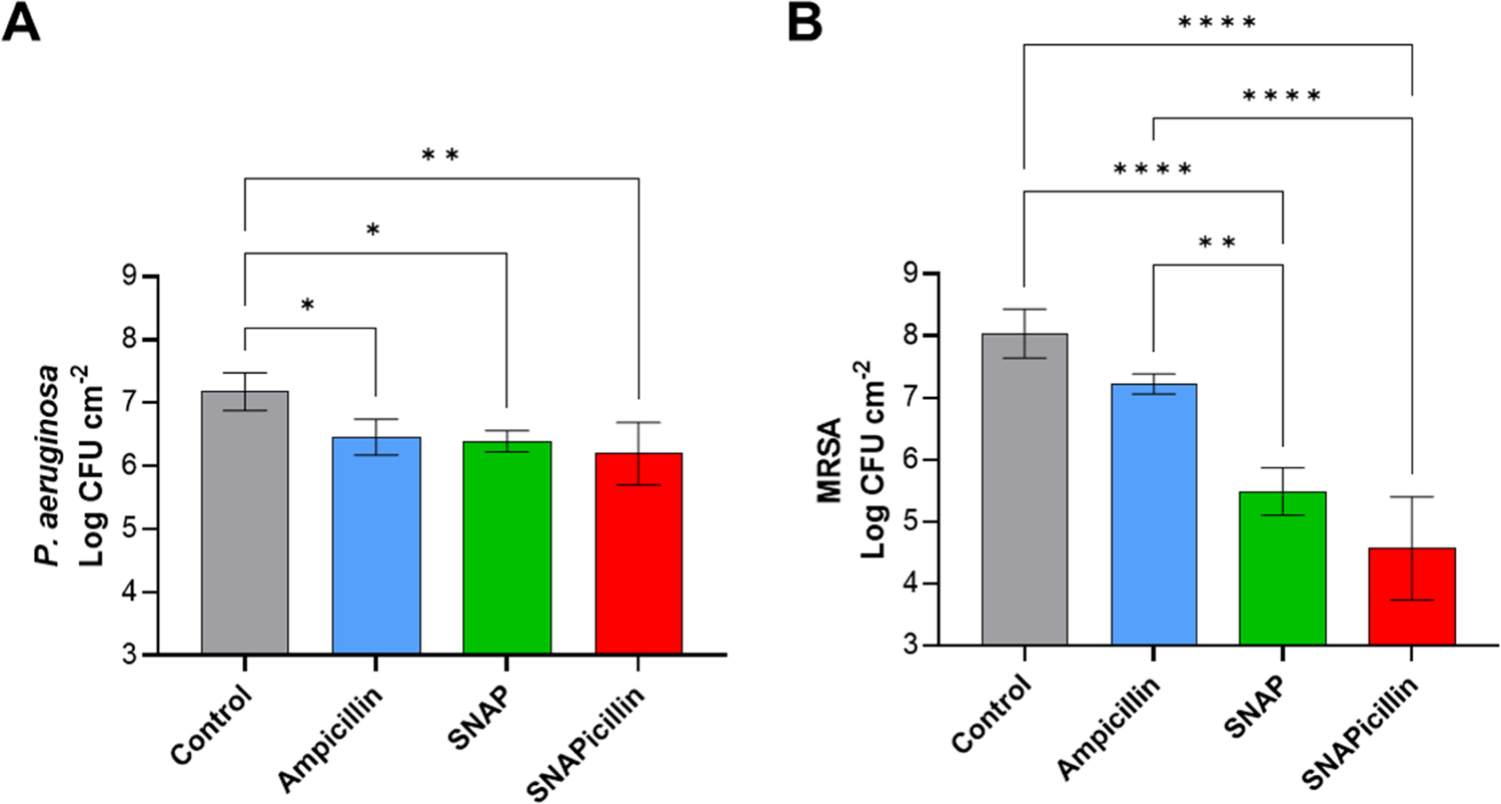
Reduction of (A) P. aeruginosa and (B) MRSA biofilms grown on CarboSil films for 48 h before treatment with ampicillin, SNAP, and SNAPicillin. Statistical significance is denoted as * (*p* < 0.05), ** (*p* < 0.01), and **** (*p* < 0.0001).

To study the effectiveness of SNAPicillin against preformed biofilms, *P. aeruginosa* and MRSA biofilms were grown for 48 h on CarboSil polymer films to mimic mature infection of a polymeric medical device. Treatment of the biofilms with ampicillin, SNAP, and SNAPicillin revealed significant differences in antibacterial effectiveness. Based on the MIC_50_ values determined in the earlier study, *P. aeruginosa* biofilms were treated with 250 μM solutions of each of the antibacterial agents. SNAPicillin showed the greatest antibacterial efficacy in terms of percent reduction, with 86.5 ± 15.7% reduction of CFUs compared to controls (Figure 5A). However, SNAP showed comparable reduction at 85.93 ± 4.70%, with no statistical significance between the two treatments.

MRSA biofilms were similarly treated with the MIC_50_ concentration of SNAPicillin found in the planktonic studies, 2 mM. In this case, SNAPicillin showed a drastic decrease in viable CFUs, with a 99.92 ± 0.10% reduction compared to controls (Figure 5B). The higher treatment concentration can explain the greater antibacterial success of SNAPicillin compared to *P. aeruginosa* biofilms. Further, we have shown in previous studies that NO has displayed greater antibacterial effects against Gram-positive bacteria compared to Gram-negative due to the extra lipopolysaccharide outer membrane that is not present in Gram-positive strains.^12,47,49^ However, when compared to treatment with ampicillin and SNAP alone, the benefit of the combination treatment is effectively demonstrated in colony counting and SEM images of treated biofilms, as NO works to break down biofilm EPS, and ampicillin and NO together are then able to eradicate the infectious pathogens. PBS (control)- and ampicillin-treated films display vast biofilm formations, while SNAP- and SNAPicillin-treated surfaces reveal single-layer bacteria coverage in conjunction with cell debris from lysed bacterial membranes and damaged internal components (Figure S4). Based on both bacteria studies, SNAPicillin is more advantageous against planktonic bacteria compared to ampicillin and SNAP. However, when treating biofilms, it behaves more similarly to SNAP due to the greater effectiveness of NO gas against resilient biofilm EPS compared to treatment with the bulky ampicillin molecule.

### 3.4 Biocompatibility Evaluation of SNAPicillin

**Figure 6:**
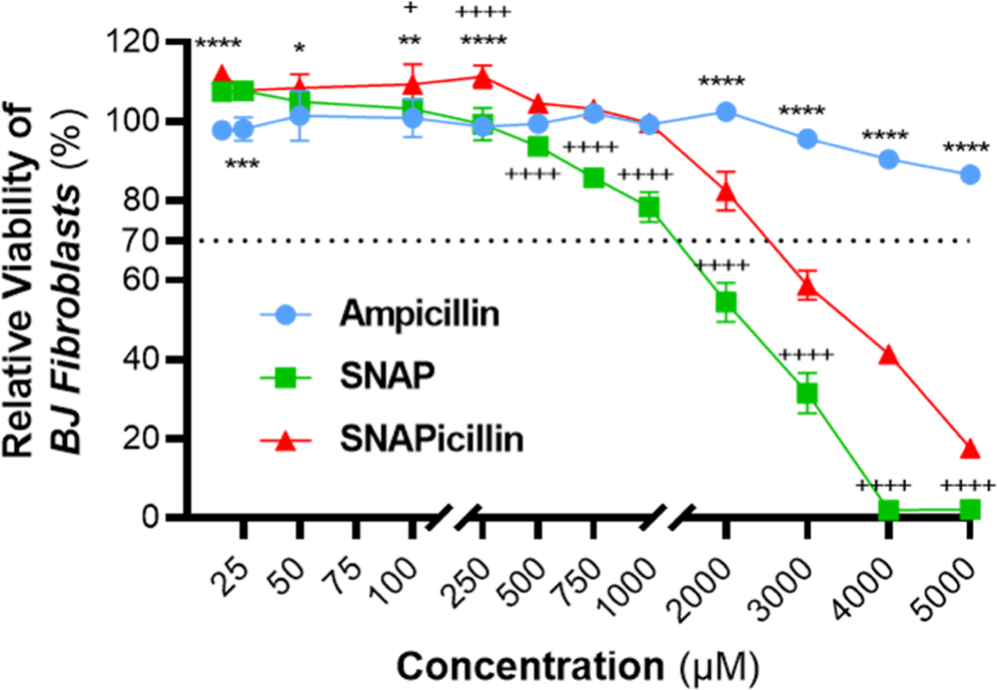
Mammalian cell biocompatibility of SNAPicillin was investigated at the effective antimicrobial concentrations and compared to ampicillin and SNAP. Final data are reported as mean relative cellular viability ± SD (*n* = 3 independent passages). Statistical significance between ampicillin and SNAPicillin at each concentration is denoted as *(*p* < 0.05), **(*p* < 0.01), ***(*p* < 0.001), and ****(*p* < 0.0001). Statistical significance between SNAP and SNAPicillin at each concentration is denoted as ^+^(*p* < 0.05) and ^++++^(*p* < 0.0001).

The antimicrobial effects of antibiotics must be critically viewed against their general cytocompatibility to ensure that any clinical application does not incur inflammatory or other off-target effects. To this end, the cytocompatibility of SNAPicillin was screened against both ampicillin and SNAP in a series of studies with human fibroblast cells in accordance with ISO-10993-5 standards.^37^ Generally, higher concentrations of SNAPicillin induced a greater cytotoxic effect against mammalian cells, likely attributable to any inherent cytotoxicity of the ampicillin backbone as well as the accumulation of NO from the decomposition of the RSNO pendant group (Figure 6). Interestingly, at lower concentrations of SNAPicillin, a general increase in cellular viability compared to ampicillin was observed (*p* < 0.05), likely attributable to low concentrations of NO inducing cellular proliferation. This effect has previously been reported with several NO-releasing polymeric materials^12,42,50^ and further supports the implementation of SNAPicillin in controlled release platforms such as polymeric materials, hydrogels, and nanoparticle formulations.

## 4 Conclusions

In summary, we have successfully modified a commonly used antibiotic, ampicillin, with NO-releasing capabilities utilizing RSNO chemistry for the first time. The novel molecule, SNAPicillin, exhibits stable concentration-dependent NO release upon exposure to physiological conditions. Fabrication of SNAPicillin was confirmed using NMR and FTIR analyses, and the conjugation efficiency and nitrosation efficiency were characterized using Ellman’s test and chemiluminescent NO detection methods, respectively. Minimum inhibitory concentration studies revealed that 250 μM and 2 mM of SNAPicillin induced >50% planktonic bacterial killing against Gram-negative *P. aeruginosa* and Gram-positive MRSA. Further, planktonic bacterial treatment with SNAPicillin showed the lowest MIC_50_ values compared to treatment with ampicillin and SNAP alone. Furthermore, when investigating anti-biofilm efficacy, SNAPicillin treatment of preformed 48 h biofilms of *P. aeruginosa* and MRSA showed greater biofilm penetration and bacterial killing than control, ampicillin, and SNAP treatments. Lastly, biocompatibility analysis against human fibroblast cells revealed no cytotoxicity concerns for antimicrobial concentrations of the molecule. Therefore, this work presents a multifunctional antimicrobial molecule with combined NO and antibiotic moieties for enhanced biofilm dispersal and bacterial eradication, with potential for polymeric incorporation for the prevention and treatment of medical device-related antimicrobial-resistant infections.

## Supporting Information Available

Carbon NMR spectroscopy of ampicillin and SNAPicillin; table of nitric oxide release from SNAP and SNAPicillin; biological replicates of MIC study against *P. aeruginosa*; biological replicates of MIC study against MRSA (PDF)

## Notes

Hitesh Handa is a co-founder and maintains a financial interest in Nytricx, Inc., a company that is investigating nitric oxide as a biomedical therapeutic for medical devices.
