# Supplementary material for: Synthesis and Characterization of Nitric Oxide-Releasing Ampicillin as a Potential Strategy for Combatting Bacterial Biofilm Formation
Source: ACS Appl Mater Interfaces. 2023 Mar 16;15(12):15185–94. doi: 10.1021/acsami.3c00140 (PMC10064314; doi:10.1021/acsami.3c00140)
Supplement: Supplementary file 1 — am3c00140_si_001.pdf [file am3c00140_si_001.pdf]

Supporting Information:

Synthesis and Characterization of Nitric Oxide-Releasing Ampicillin as a Potential Strategy for Combatting Bacterial Biofilm Formation

Lori M. Estes Bright[a], *Mark R. S. Garren*[a], *Megan Douglass*[a], *and Hitesh Handa*[ab*]

[a] School of Chemical, Materials and Biomedical Engineering, University of Georgia, Athens, GA 30602, United States

[b] Pharmaceutical and Biomedical Sciences Department, College of Pharmacy, University of Georgia, Athens, GA 30602, United States

[*]Corresponding Author

Hitesh Handa, Ph.D.

Associate Professor

University of Georgia

302 East Campus Rd.

Athens, GA 30602

Telephone: (706) 542-8109

E-mail: hhanda@uga.edu

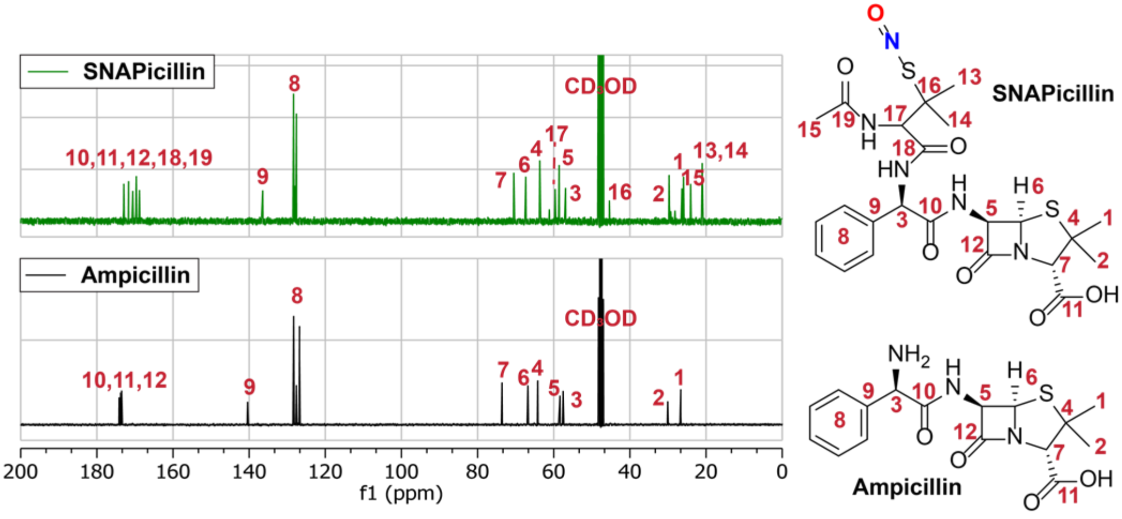

Figure S1. *Carbon NMR Spectroscopy of ampicillin and SNAPicillin following modification.*

Table S1. *Nitric oxide release from SNAP and SNAPicillin in equimolar concentrations over 24 h*

	**NO Release h0 ($x10^{-10}$ mol min^{-1} mL^{-1})**	**NO Release h24 ($x10^{-10}$ mol min^{-1} mL^{-1})**
8 mM SNAP	29.25 ± 3.6	19.17 ± 2.8
8 mM SNAPicillin	15.94 ± 1.7	13.41 ± 1.8
2 mM SNAP	7.92 ± 1.2	3.94 ± 0.8
2 mM SNAPicillin	3.72 ± 0.6	1.79 ± 0.4
500 µM SNAP	1.78 ± 0.2	0.89 ± 0.1
500 µM SNAPicillin	1.19 ± 0.3	0.52 ± 0.1
125 µM SNAP	0.44 ± 0.1	0.19 ± 0.05
125 µM SNAPicillin	0.53 ± 0.05	0.18 ± 0.01

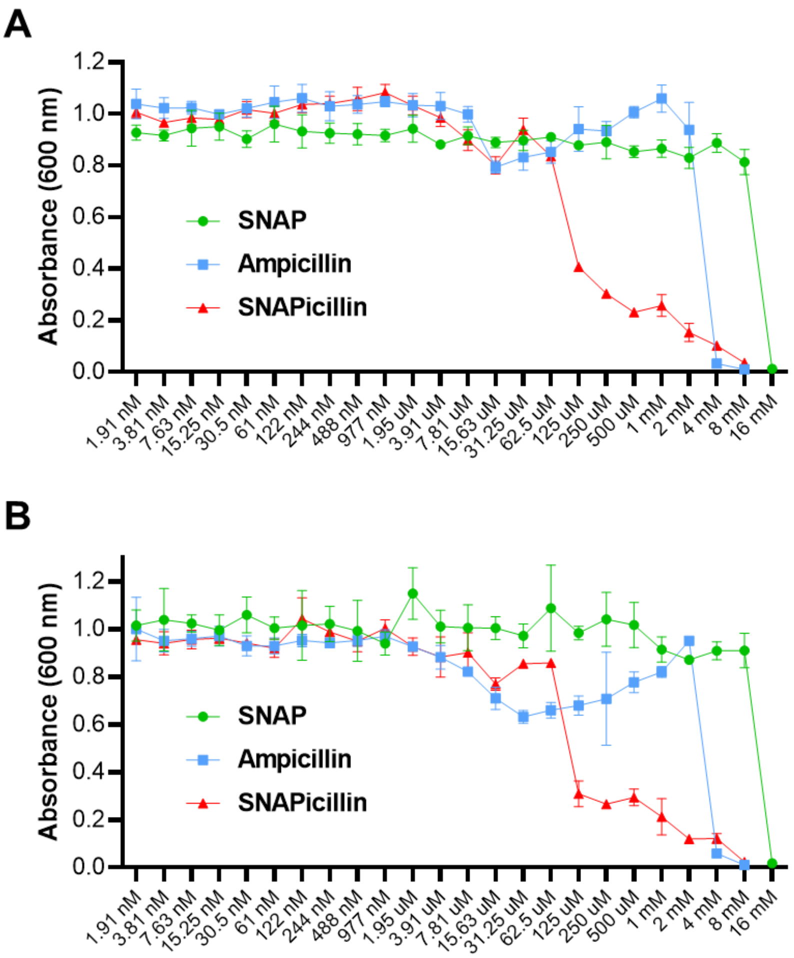

Figure S2. *Biological replicates of MIC study against P. aeruginosa.*

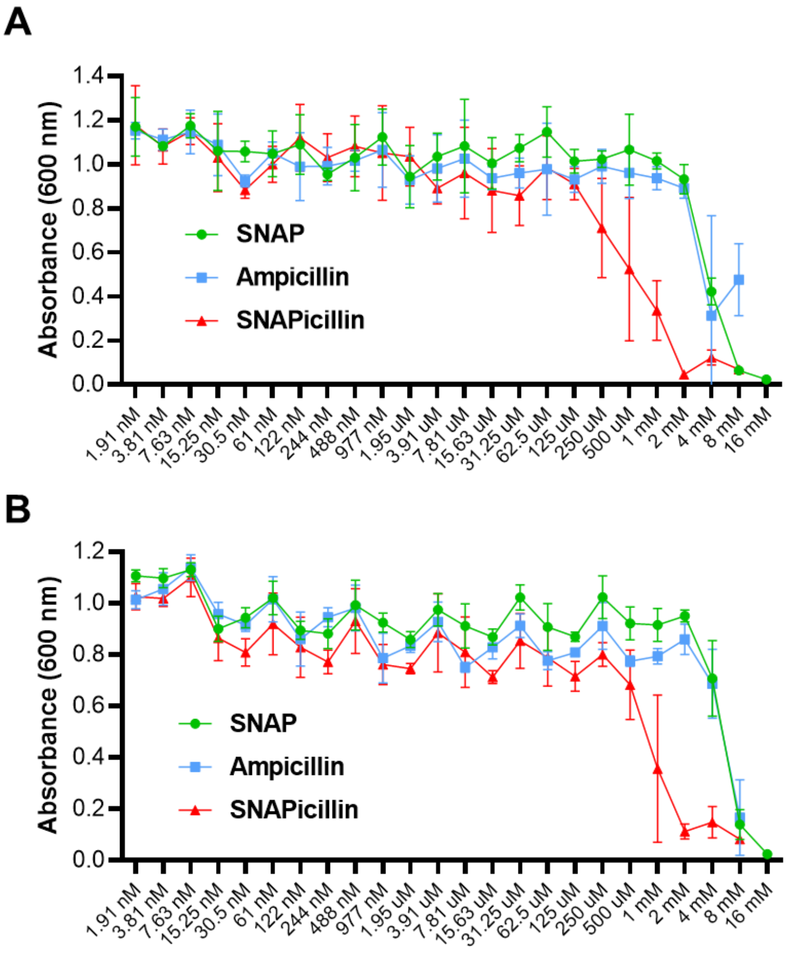

Figure S3. *Biological replicates of MIC study against MRSA.*

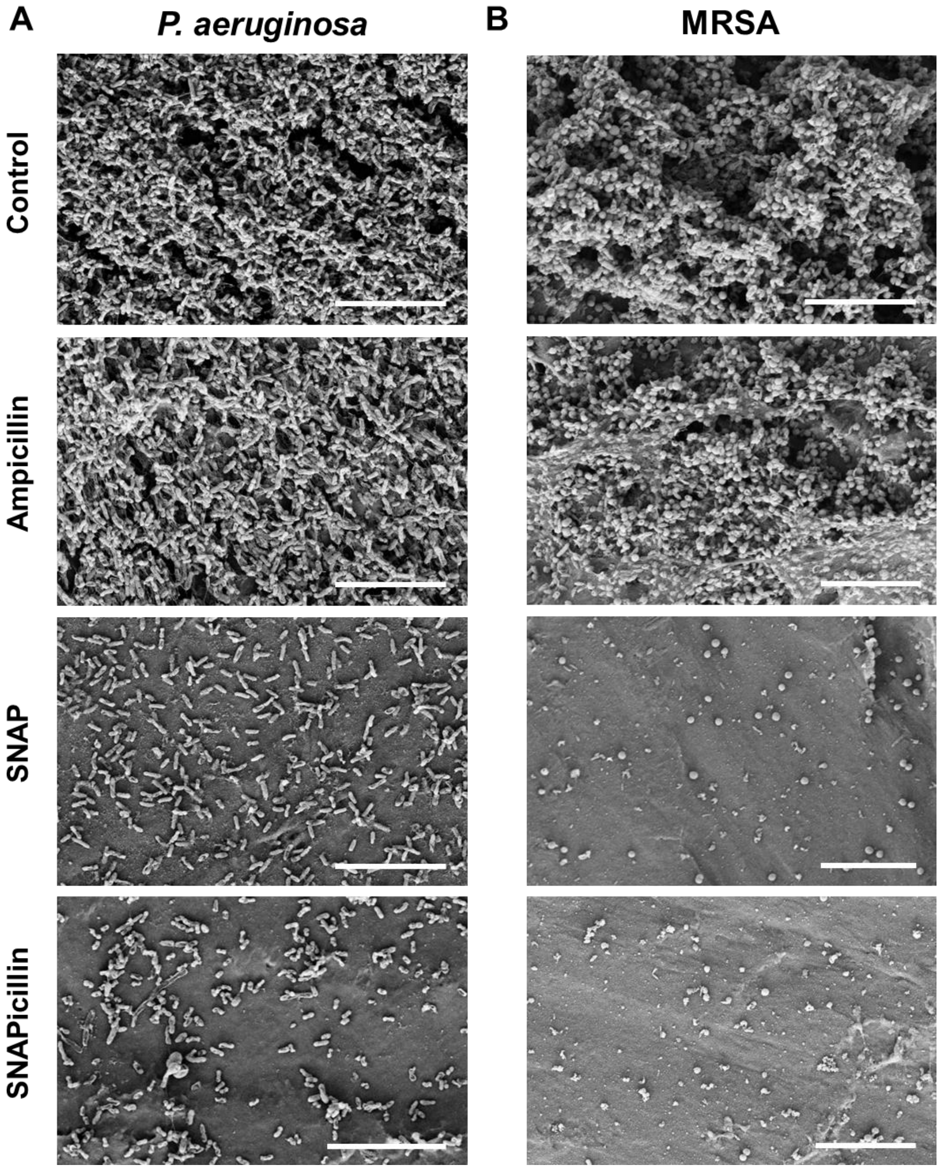

Figure S4. *SEM images of biofilm dispersal following treatment of 48 h preformed biofilms with PBS (control), ampicillin, SNAP, and SNAPicillin.*
